# aCGH Analysis to Estimate Genetic Variations among Domesticated Chickens

**DOI:** 10.1155/2016/1794329

**Published:** 2016-07-21

**Authors:** Tomoyoshi Komiyama, Mengjie Lin, Atsushi Ogura

**Affiliations:** ^1^Department of Clinical Pharmacology, Tokai University School of Medicine, 143 Shimokasuya, Isehara, Kanagawa 259-1193, Japan; ^2^Department of Biology, Ochanomizu University, Tokyo 112-0012, Japan; ^3^Nagahama Institute of Bio-Science and Technology, Nagahama, Shiga 526-0829, Japan

## Abstract

Chickens have been familiar to humans since ancient times and have been used not only for culinary purposes but also for cultural purposes including ritual ceremonies and traditional entertainment. The various chicken breeds developed for these purposes often display distinct morphological and/or behavioural traits. For example, the Japanese* Shamo* is larger and more aggressive than other domesticated chickens, reflecting its role as a fighting cock breed, whereas Japanese* Naganakidori* breeds, which have long-crowing behaviour, were bred instead for their entertaining and aesthetic qualities. However, the genetic backgrounds of these distinct morphological and behavioural traits remain unclear. Therefore, the question arises as to which genomic regions in these chickens were acted upon by selective pressures through breeding. We compared the entire genomes of six chicken breeds domesticated for various cultural purposes by utilizing array comparative genomic hybridization. From these analyses, we identified 782 regions that underwent insertions, deletions, or mutations, representing man-made selection pressure in these chickens. Furthermore, we found that a number of genes diversified in domesticated chickens bred for cultural or entertainment purposes were different from those diversified in chickens bred for food, such as broilers and layers.

## 1. Introduction

Today, many chicken breeds have been kept worldwide as laying hens and for poultry, as well as for other purposes or as pets. However, chicken domestication extends back to antiquity, when the chicken was domesticated to provide meat and eggs, which are valuable culinary items [1–3], and to perform various other tasks. For example, as chickens crow loudly at dawn, they were used for reporting the time. In some regions and societies, the chicken was considered a mysterious animal with a beautiful appearance and song, and humans enthusiastically bred them to suit a more ritual role. In Japan, fighting cocks and long-crowing chickens are typical examples of chickens that have been bred for entertainment and aesthetics. Past studies regarding the molecular evolution of these chickens in Asia revealed that cultural domestication has imposed a strong artificial selective pressure [4]. Moreover, phylogenetic analyses of these chickens clarified that their domestication process was tightly connected to Japanese culture [5, 6].

Thus, people have improved the desirable characteristics of chickens to make use of them in daily life. The origin of all modern domesticated chicken breeds is considered to be the red junglefowl (*Gallus gallus*), one of four species of wild fowl indigenous to Southern China, Southeast Asia, and parts of Southwest Asia [7]. The domestication of chickens is believed to have started in the Indus Valley region around 2000 BC [8]; however, based on archaeological evidence West and Zhou [9] argued that much earlier domestication arose in mainland China around 6000 BC. The genetic changes that accumulated during the domestication process of broilers and layers are an important target for population genetics and molecular evolution as well as for animal science and stockbreeding. To study the domestication of chickens for cultural purposes rather than for food purposes is important not only for clarifying the genomic backgrounds of “culturally domesticated” chickens but also for elucidating differences in genomic changes and in artificial selection among various breeds for diverse purposes.

In this study, we focused on genetic diversity among culturally domesticated chickens in Japan, which contains many domesticated chicken breeds. Each of these breeds is characterized by a different temperament, physical shape, and behaviour, attained through specialized breeding and artificial selection. For example, fighting cocks, commonly called* Shamo*, have been bred solely for the purpose of cockfighting. Likewise, long-crowing chickens, commonly known as* Naganakidori*, have been specifically bred to acquire an exceptionally long crow of over 15 seconds [5]. Similarly, the* Chabo* and* Minohikidori* were bred for ornamental purposes through a special process [10].

**Figure 1:**
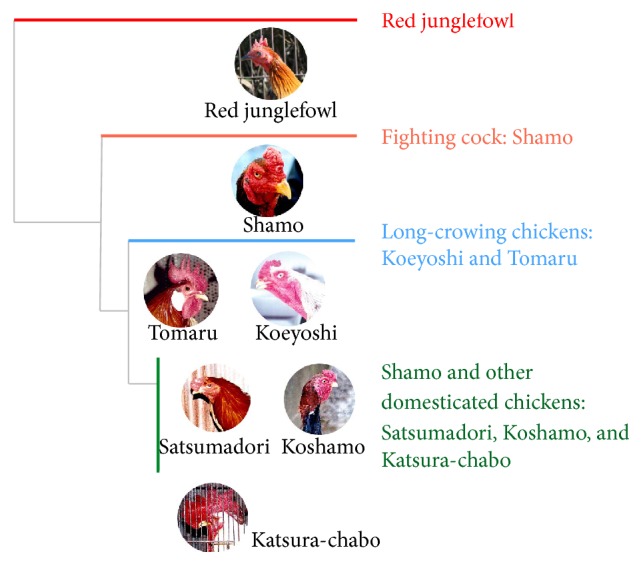
Phylogenetic tree of wild and culturally domesticated chickens used in this study. The phylogenetic tree was drawn using nucleotide sequences of mitochondrial D-loop regions of our previous result [6].

Japanese domesticated chickens are excellent models for studying the influence of human culture on animal domestication and breeding. We previously found that numerous intense artificial selection events occurred before the divergence of Japanese chickens from ancestral fighting cocks, as suggested by the remarkably different phenotypes of Japanese ornamental chickens [5, 6, 10, 11]. Further studies of domesticated and wild chickens in Asia using mtDNA and nuclear DNA sequences as well as the domestication process of modern chickens have remained unexplored. In our molecular evolutionary studies based on the mitochondrial D-loop region, the fighting cock breed* Shamo* was found to have first diverged from red junglefowl,* G. gallus*, and then later converged to form clusters, long-crowing chicken breeds* Koeyoshi* and* Tomaru* group and then the* Shamo*,* Katsura-chabo*,* Satsumadori*, and* Koshamo* group (Figure 1) [6]. These Japanese-bred domesticated chickens are descendant of* G. gallus gallus* and can be interbred.

In 2004, Wong et al. reported their study of whole-genome single nucleotide polymorphisms (SNPs) to uncover genetic variations in chicken. Furthermore, Muir et al. [12] reported the significant absence of rare alleles in commercial breeds by genome-wide SNP assessment. Genome-wide surveys for SNPs and quantitative trait loci related to chicken domestication have been conducted by several other groups [13, 14] but no studies examined differences among breed characteristics. In recent research, Rubin et al. [15] expanded this approach by resequencing the whole genome to reveal loci under selection during the domestication of chickens for food; however, they found little evidence that selection for loss-of-function mutations had a prominent role in chicken domestication. Only a few studies have shed light on the genetic variations in various chickens [16, 17]. There are several other studies focusing on domestication itself, but none of this research has focused on the domestication aimed for cultural purposes [18–21].

Against this background, the objective of this study is to understand the process of the cultural domestication of chickens by identifying and characterizing the genetic factors that have contributed to the phenotypic changes from ancestral wild fowl (i.e., red junglefowl) to domesticated chickens, such as behaviour, body size, and comb type. We examined chickens bred for fighting and as ornamentals by array comparative genomic hybridization (aCGH) to identify genes highly specific to these breeds. The aCGH is a method of identifying genetic variations among samples by utilizing genomic DNA and a microarray. Our aCGH is designed to investigate genetic variations within groups of culturally domesticated chickens.

## 2. Materials and Methods

### 2.1. Sample Collection

We obtained a total of 7 samples, fighting cocks (*Shamo* 49 and* Satsumadori* 31) [6], long-crowing chickens (*Naganakidori*:* Tomaru* 203 and* Koeyoshi* 27), other ornamental chickens (*Katsura-chabo* 20 and* Koshamo* 13), and one Yakei (*G. gallus* 222), from the Bird Center of Kurume in Fukuoka Prefecture. These domesticated chickens had been collected in our previous studies. In addition, these numbers are linked to our previous researches [4–6, 11].

### 2.2. Probe Design and aCGH

A total of 59,801 probes, representing 17934 genes, were designed using the Chicken HD probe set in eArray (Agilent Technologies, Santa Clara, CA, USA). Two sets of 8x60K arrays were used for the aCGH experiments. A genomic DNA labelling kit was used to label gDNAs that were hybridized to the chicken CGH array. The Yakei was used as a reference sample, and six culturally domesticated chickens were used in aCGH experiments. Dye-swap experiments were also performed.

### 2.3. Statistical Analysis

We removed probes that were flagged as either “not uniform” or “population outlier.” We then removed control probes and unreliable data that were inconsistent with the results of the dye-swap experiments and obtained 47,308 probes out of 59,801 probes. We then classified probes into three categories: (1) probes with intensities less than 0.5x median of all probes; (2) probes with intensities between 0.5 and 2.0x of the median of all probes; and (3) probes with intensities larger than 2.0x of the median of all probes. One-way ANOVA was performed under the following conditions: *p* value computation; Asymptotic Multiple Testing Correction; Benjamini-Hochberg FDR; and Number of Permutations, 100. We ultimately obtained 6,385 statistically significant probes with a corrected *p* value of 0.05. Fold change: we first collected probes that matched both the probes that were not changed in the Yakei (42,175 probes) and the probes that were selected by ANOVA (6,385 probes). We then extracted probes in which the probe intensity was either 2.0x larger than that in the Yakei or 0.5x less than that in the Yakei in each strain.

## 3. Results

### 3.1. aCGH Analysis to Estimate Genetic Variations among Culturally Domesticated Chickens

**Figure 2:**
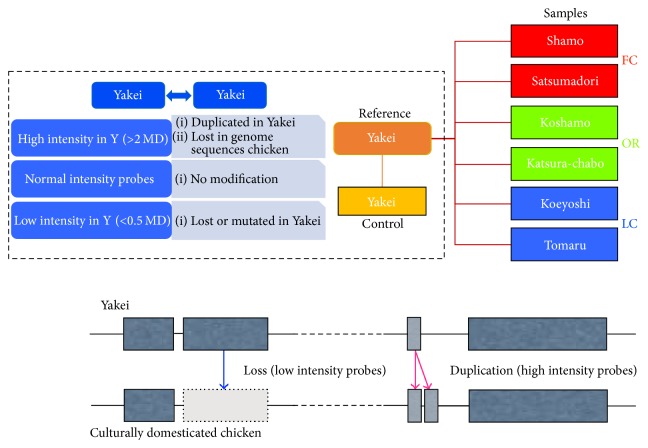
Experimental design and analytical scheme. To unveil genetic variations between Yakei (Y) and genome sequenced chicken (GSC). In the aCGH experiments, hybridizations were performed using Y on a custom microarray in which probes were designed from GSC. To clarify genetic variations in culturally domesticated chickens Y (reference) and 6 domesticated chickens (sample) were used for aCGH experiments.

**Figure 3:**
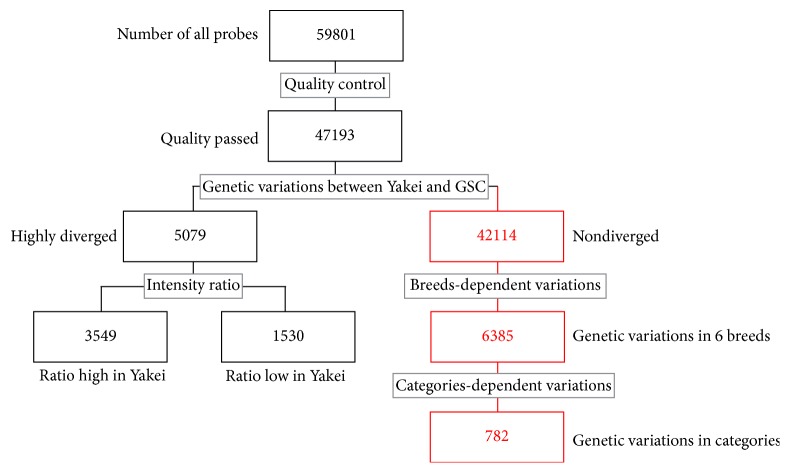
Scheme for the experiment and analysis. Figures indicate the number of probes after analyses shown in grey boxes.

To understand genetic variations underlying cultural domestication of chickens, aCGH analysis has been performed employing a microarray designed from the genome of the classic genome-sequenced domesticated chicken (GSC) and hybridization experiments using six culturally domesticated chickens, comprising two fighting cock breeds, two long-crowing chickens breeds, and two ornamental chickens breeds, as well as the red junglefowl, as the reference genomic DNA. We used the eArray (Agilent Technologies, Santa Clara, California) for probe design and made two array slides with 8x60K probes that would cover all the chromosomes of the chickens. We then performed two-colour hybridization with dye-swap using gDNA from six culturally domesticated chickens as well as from Yakei as a control (Figure 2). Schematic workflow of this study is shown in Figure 3.

**Figure 4:**
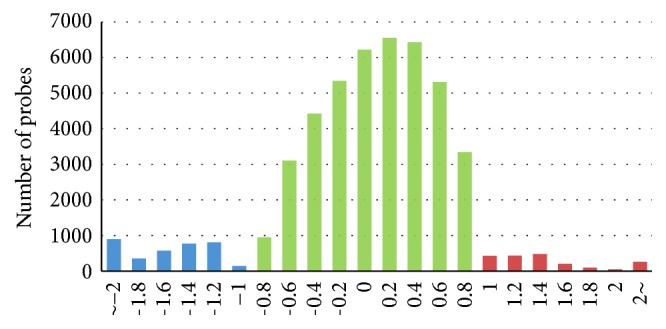
Overall genetic variations in culturally domesticated chickens. Regions with relative intensity between samples and the control less than −1.0 were categorized as “lost or mutated candidates in Yakei (Y)” (blue colour). Regions with relative intensity between samples and the control larger than 1.0 were categorized as “duplicated candidates in Yakei (Y)” (red colour). Nondiverged probes indicated in green colour. The *y*-axis represents fold changes on a log2 scale.

To estimate genetic variations among culturally domesticated chickens, we first extracted the genetic variations derived from human breeding. We then compared the array results of the Yakei (reference) and the GSC (control) (Figures 2 and 3). As our aCGH is designed from the GSC, it is essential to distinguish the genetic variation during cultural domestication (one Yakei to six domesticated chickens) from the genetic variation that has already been accumulated in the branch of GSC to Yakei. We first removed unreliable results from the array data using GeneSpring and obtained 47,193 probes, among which 42,114 probes were considered to be not diversified from those of the GSC. Among the remaining 5,079 probes, 3,549 had higher intensity than the median intensity of all probes, which would be accounted for by one of the following three explanations: (1) corresponding genomic regions were lost or highly mutated in the Yakei lineage, (2) corresponding genomic regions were lost or highly mutated in the GSC, or (3) corresponding genomic regions were duplicated in the GSC (Figure 4). To distinguish between these possibilities for each probe, we examined duplicated regions or corresponding genomic regions against the rest of the genome sequence using electronic polymerase chain reaction (PCR) and found that 1,522 regions had been duplicated in the GSC and that the remaining probes had not undergone any duplication events. The remaining 1,530 probes out of the 5,079 diversified probes had more than double the median intensity of all probes, implying that those regions might be duplicated in the Yakei lineage. These results suggest that 10.8% (5,079/47,193) of the regions in the chicken genome have been altered in the branch of the GSC and Yakei (Figure 4).

**Figure 5:**
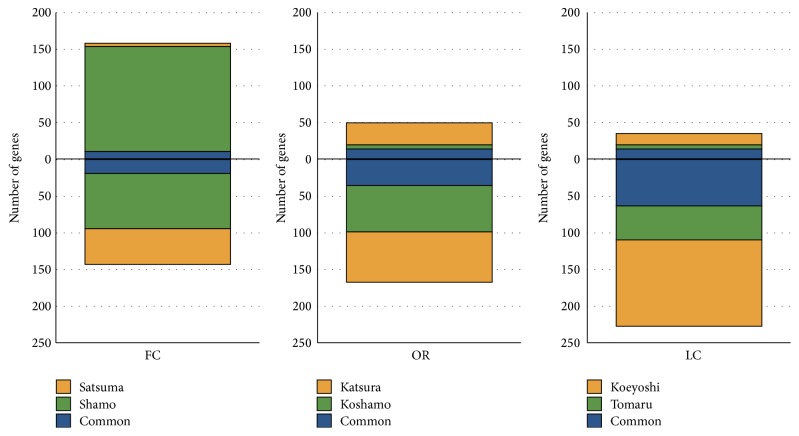
Duplicated genes and mutated or lost genes in the fighting cocks (FC), ornamental chickens (OR), and long-crowing chickens (LC). The upper half indicates the number of duplicated genes in the categories; colour indicates common or strain specific genes in each chicken breed. The lower half indicates the number of lost or mutated genes in the categories.

To investigate genetic variations in culturally domesticated chickens, we analysed aCGH data for Yakei and six such breeds:* Shamo* (fighting cock),* Satsumadori* (fighting cock),* Tomaru* (long-crowing),* Koeyoshi* (long-crowing),* Katsura-chabo* (ornamental), and* Koshamo* (ornamental) (Figure 2). To estimate the genetic alteration in these six breeds, we utilized a total of 42,114 probes for further analysis that were not diversified in GSC to Yakei branch (Figure 3). Next, 6,385 of the 42,114 probes were further selected as candidate regions for genetic diversification in any of culturally domesticated chickens by one-way ANOVA. The gene set enrichment analysis of 6,385 probes was then performed. The gene ontology terms associated with “cell periphery” and “signaling processes” were significantly overrepresented in these probes. In all breeds except the* Shamo*, the number of lost or mutated candidates exceeded those of duplicated candidates, especially in* Koeyoshi*. Concerning domesticated category-specific variations, 782 probes have been found in fighting cocks, long-crowing chickens, and ornamental chickens that differ by a fold-change threshold of 2.0 (Figure 4). These results indicated that the fighting cocks tended to duplicate genes during domestication for the purpose of combat, as opposed with the ornamental (OR) and long-crowing (LC) chickens, which tended to have a higher proportion of mutated or lost genes. In addition, two breeds of the fighting cock (*Shamo* and* Satsumadori*) had fewer genes in common (blue) compared with the breeds in other categories (Figure 5), because the fighting cock group was composed of relatively distant lineages as shown in Figure 1. To validate the estimation of genomic variations, we amplified 26 among 782 candidates by PCR based on the primer designed from sequence obtained from GSC. As a result, 16 out of 26 regions were confirmed to be mutated in the corresponding chickens (see Table 1) (Supplementary Data 1 in Supplementary Material available online at http://dx.doi.org/10.1155/2016/1794329).

### 3.2. Genes Subjected to Selection Pressures under Cultural Domestication

Next, we searched for candidate genes that may have been subjected to selection pressures under cultural domestication. We found that even though most candidates were lost regions, the* Shamo* breed and* Naganakidori* type breeds possess a large number of candidate duplicated regions, most of which are related to the nervous system and membrane proteins. Therefore, regions duplicated in* Shamo* are related to muscle development genes, such as* MYH1* and* MYH7B* [22–24]. We have also found genes as candidates, namely,* IGF-2*,* Robo1*, and* DCX* [25–35]. These regions might have undergone human selection pressure for fighting ability. We have validated that* IGF1*,* MYH1*, and* MYH7B* genes were duplicated in different chromosomes that might affect gene expression efficiency at the level of muscular development.

The* Koeyoshi,* long-crowing chicken breed, has lost many genes common to other cultural domesticated chickens. In addition,* Katsura-chabo* was revealed to possess interesting genes. In particular,* PIT1* genes were associated with chicken growth traits [36–38]. Compared to wild fowl, domesticated chickens are smaller, and the male grows to only approximately 1.0 kilograms in weight [10].* Koeyoshi* and* Chabo* also have undergone strong artificial selection by ancient humans. Although gene expression analyses for differences among commercially domesticated chickens are intensively performed [21, 39–41], none of the above genes have been reported. Therefore, these genes are thought to be related to cultural domestication rather than domestication for commercial purposes.

### 3.3. Comparison of Genetic Changes between Cultural Domestication and Food Domestication

aCGH analysis allowed us to obtain 782 probe candidates for cultural domestication in long-crowing, fighting, and ornamental breeding lines (Figure 3). We then compared our “cultural domestication genes” with “food domestication genes” that have been reported as genetic variations in food domestication lines [15]. We first selected 589 genes corresponding to 782 probes and then searched common genes with 540 food domestication genes. We found that 28 genes had been commonly but independently changed in both culturally domesticated chicken lines and food domesticated chicken lines. To test the significance of the representation of overlap genes between the different domestication lines, we computed *p* values using Fisher's exact test and found no correlation. In other words, the target of selection pressure was not conserved between different breeds. We also checked all selective sweep sites identified by Rubin et al. [15] and also confirmed that the genetic variation loci for cultural and food domestication are independent.

## 4. Discussion

Various breeds of chicken are kept worldwide as pets or for meat, eggs, or other purposes. It is believed that all chicken breeds originated from the red junglefowl, one of four species of wild fowl indigenous to Southern China, Southeast Asia, and part of Southwest Asia [7]. Over the course of domestication, the red junglefowl has evolved into a variety of breeds. However, it remains unknown which factors have driven the change from the ancestral species to the currently established chicken breeds.

It is an undeniable fact that human interests strongly influenced the establishment of different chicken breeds during domestication of wild fowl. For example, the bird might have been used in rituals as it was considered by ancient people to be a mysterious animal with a beautiful appearance and song. It is also highly possible that people took advantage of the time-reporting characteristics of the bird, as it crows loudly at dawn, in their daily life. There is also no doubt that domestication focused on providing poultry and eggs, which are readily available culinary items [1–3].

It is worth mentioning that some domesticated Japanese chickens have existed simply for appreciation since the Edo period (1601–1867) and are now considered national treasures. In particular, ancient Japanese people began breeding their favourite* Shamo* varieties more than 1,000 years ago [11].

The objective of this study was to characterize the ongoing process of chicken domestication by comparative genomic hybridization array analysis, to identify the genetic factors that have contributed to the change from wild fowl to domestic chicken, to identify genes highly specific to chickens for ornamental, fighting, and food purposes, and to determine the sequence of these genes. In recent research, Rubin et al. whole-genome resequencing revealed loci under selection during food chicken domestication, which found little evidence that selection for loss-of-function mutations had a prominent role in this type of domestication (in White Leghorn, Rhode Island Red, Minorca, etc.), but they detected two deletions in coding sequences that they suggested are functionally important.

From our results, category-specific variations were made. We found 782 probes with a fold-change threshold of 2.0 in fighting cocks, long-crowing chickens, and ornamental chickens. To validate the estimation of genomic variations, we amplified 26 of the 782 candidates via PCR based on a primer designed from sequences obtained from the GSC. As a result, 16 of the 26 regions were confirmed to be mutated in the corresponding chickens.

In our analysis of the aCGH results, we estimated the existence of more than 600 probe candidates for genes undergoing human selection pressure in culturally domesticated chickens. We also found that a number of genes diversified in culturally domesticated chickens are different from those diversified in chickens domesticated for food, such as in broilers and layers.

More interestingly, variations in* Shamo*, bred for cockfighting, were found to be related to muscle development genes, such as* MYH1* and* MYH7B*. Fighting cocks have always been selected for strength, and this selection pressure is responsible for the high number of crosses. Therefore, the* Shamo*, which was bred for cockfighting, has a muscular, broad body and conspicuous appearance. The* Shamo* has been bred intensively in Japan, although the tradition of cockfighting is distributed worldwide. Here, we found different specific genes developed for cockfighting between the* Shamo* and* Satsumadori* breeds for cockfighting breeds. In general, the* Shamo* and* Satsumadori* were bred by ancient humans for different traits, reflecting different regional styles of cockfighting. One style is similar to boxing and the other is more similar to fencing and uses attached blades [42, 43]. These different rules have occurred in different regions of the globe, causing variation of genes developed within breeding chickens for each style of cockfighting.

Additionally, the* Koeyoshi*, long-crowing chicken breed, has lost many genes that are common in other culturally domesticated chickens. The life expectancy of* Koeyoshi* with good voices is short, only approximately two years [4]; however,* Koeyoshi* with weak voices have a much longer life expectancy of up to four years. A strong artificial selection is likely responsible for the shortened life expectancy of* Koeyoshi*. The specific genes found in this study might have related to its short life. In addition, the results in the small* Katsura-chabo* revealed that the* PIT1* gene was associated with chicken growth traits. Using aCGH, we could find each specific gene acted upon strongly by artificial selection in culturally domesticated chickens. Therefore, these specific genes are highly relevant for various fields of ongoing research in domesticated chickens or for the domestication process to preserve these breeds in the future.

Humans have improved the desirable characteristics of chickens to make use of them in daily life. In the same way, chickens have taken advantage of their surroundings and characteristics and have continued to change in appearance and nature to leave behind offspring that is coexisting with humans.

## 5. Conclusions

We examined the genes that are responsible for aggressiveness and crowing behaviour by utilizing aCGH analysis with six breeds of culturally domesticated chickens representing fighting cocks, long-crowing chickens, and ornamental chickens. From our analysis of the aCGH results, we estimated the existence of 782 probe candidates for genes undergoing human selection pressure in culturally domesticated chickens. Upon comparing a wild variant, Yakei, and the GSC chicken that has been fully sequenced, 10.9% of genomic regions were found to have been modified. Growth hormone-related genes known to be modified in food domesticated lines were also genetically modified in long-crowing chickens. We also found that a number of genes diversified in culturally domesticated chickens are different from those diversified in chickens, such as broilers and layers, which have been domesticated for food.

## Supplementary Material

CR validation of Duplicated or lost genes found with aCGH analysis.
We have selected four or five genes from the Koeyoshi genes (lost or highly mutated), Shamo genes (duplicated/lost or highly mutated) and Katsura-chabo (lost or highly mutated). Probe names, corresponding Ensembl gene names and annotations, and primers as shown in the table.

## Figures and Tables

**Table 1:** Enriched GO categories in the genes for human selection pressures in the domesticated chickens.

GO term	Corrected *p* value	Count in selection	Count in total
Cell periphery	0.001	204	750
Plasma membrane	0.001	204	712
Membrane	0.001	559	1,810
Membrane part	0.001	313	1,244
Signaling process	0.003	142	1,048
Signal transmission	0.003	3.5	1,048
Synapse	0.004	41	112
Plasma membrane part	0.006	27	420

GO: gene ontology.
